# Oral status associated with xerostomia and oral health-related quality of life: an older population-based cross-sectional study in China

**DOI:** 10.1186/s12877-026-07876-y

**Published:** 2026-06-24

**Authors:** Kaijing Zhong, You Wang, Yunyun Zhou, Xinyang Huang, Jing Cheng, Fuling Zhou, Bo Cheng

**Affiliations:** 1 Department of Stomatology, Zhongnan Hospital of Wuhan University, 169 Donghu Rd, Wuhan, 430071 China; 2 School of Nursing, Wuhan University, Wuhan, Hubei China; 3 Research Center for Lifespan Health, Wuhan University, Wuhan, Hubei China

**Keywords:** Xerostomia, Tooth loss, Oral health-related quality of life, Older adults, Oral health

## Abstract

**Background:**

Xerostomia is a common condition among older adults and has been associated with adverse oral health outcomes and reduced oral health-related quality of life (OHRQoL). This study aimed to investigate the associations among clinical oral health indicators, xerostomia, and OHRQoL in a community-dwelling elderly population in China.

**Methods:**

This cross-sectional study was conducted among 583 community-dwelling adults aged ≥ 60 years in Jingzhou, Hubei, China. The severity of xerostomia was assessed using the xerostomia inventory (XI), and OHRQoL was evaluated using the geriatric oral health assessment index (GOHAI). Clinical oral examinations included assessments of decayed teeth, filled teeth, missing teeth, periodontitis, and prosthetic restoration status. Logistic regression was performed to examine the association between oral health indicators and xerostomia. Linear regression was used to evaluate factors associated with GOHAI scores. Receiver operating characteristic (ROC) analysis was conducted to explore the discriminative performance of combined oral health-related indicators for differentiating participants with and without xerostomia symptoms classified by the XI.

**Results:**

The sample comprised 330 females (56.6%) and 253 males (43.4%), with a mean age of 71 years (SD = 6.4), followed by mild (39.3%), moderate (11.3%) and severe xerostomia (0.2%), respectively. Compared with participants without tooth loss, higher odds of xerostomia were observed among those with more than 10 missing teeth (OR = 1.94, 95% CI: 1.04 –3.60). Lower GOHAI scores were independently associated with xerostomia (B = -7.05, 95% CI: -8.19 to - 5.91), 6–10 missing teeth (B = -2.80, 95% CI: -4.74 to -0.86), and > 10 missing teeth (B = -3.33, 95% CI: -5.19 to -1.47). The combined model demonstrated acceptable discriminative performance (AUC = 0.720).

**Conclusion:**

Xerostomia was prevalent among community-dwelling older adults and was independently associated with greater tooth loss. Both xerostomia and advanced tooth loss were independently associated with poorer OHRQoL.

**Supplementary Information:**

The online version contains supplementary material available at https://doi.org/10.1186/s12877-026-07876-y.

## Introduction

The global population of older adults has been rising rapidly, particularly in China where recent estimates indicate that the number of individuals aged 60 years or older has reached 253 million [1]. Maintaining oral health is a critical component of healthy aging and forms a core aspect of preventing the oral frailty syndrome, which links cumulative oral disease burden to functional and psychosocial well-being [2]. Chronic oral conditions, primarily dental caries and periodontitis, remain highly prevalent among older adults and represent leading contributors to progressive tooth loss, which can impose functional limitations and impact quality of life [3] .

Xerostomia, defined as the subjective perception of oral dryness, is frequently reported in older populations and has been associated with adverse oral health outcomes, including reduced masticatory efficiency, impaired swallowing, increased susceptibility to dental erosion, and compromised oral health-related quality of life (OHRQoL) [4–8]. Emerging evidence suggests a bidirectional relationship between tooth loss and salivary function: advanced tooth loss is associated with reduced masticatory stimulation, which may correlate with lower stimulated salivary flow and contribute to subjective xerostomia [9, 10]. Conversely, persistent xerostomia may accelerate cumulative oral disease through diminished buffering capacity and impaired antimicrobial activity, increasing the risk of root caries and erosion [7, 8]. However, the independent contribution of xerostomia relative to other clinical oral health indicators, such as decayed teeth, filled teeth, periodontitis, and prosthetic restoration status, remains incompletely understood, particularly in older adults with complex lifetime exposure to dental pathology.

Previous studies have often relied on self-reported tooth counts or aggregated indices (e.g., DMFT) rather than detailed clinical examinations of individual oral health components, which may obscure the distinct contributions of tooth loss, active decay, and restorations [7–10]. Moreover, many studies fail to account for important health-related confounders, such as diabetes, that may influence salivary function and oral health outcomes. These limitations highlight a need for more comprehensive, clinically grounded investigations in representative older populations.

To address these gaps, the present cross-sectional study aimed to examine the independent associations among comprehensive clinical dental status, subjective xerostomia, and OHRQoL, as measured by the Geriatric Oral Health Assessment Index (GOHAI), in a well-characterized cohort of community-dwelling older adults in Jingzhou, Hubei, China.

## Materials and methods

### Study design and data sources

This cross-sectional study was conducted among community-dwelling participants aged 60 years or above in Jingzhou, Hubei Province, China, from May to June 2024. The study complied with the ethical principles of the Helsinki Declaration and was approved by the Ethics Committee of Zhongnan Hospital of Wuhan University. Written informed consent was obtained from all participants prior to enrolment.

A two-stage cluster sampling method was adopted to recruit participants from the older adult population. In the first stage, all six administrative districts of Jingzhou were included, and one community hospital was designated as the primary sampling units (PSUs), randomly selected from each district using a computer-generated random sampling method. In the second stage, cluster sampling was conducted within each selected hospital. Specifically, all eligible community residents aged 60 years or older who attended routine annual health check-ups or participated in community health programs at these centers between May and June 2024 were consecutively invited to participate to minimize selection bias. The inclusion criteria were as follows: (1) aged 60 years or older; (2) permanent residents of Jingzhou community; and (3) able to communicate and complete the questionnaire independently or with investigator assistance. Conversely, participants were excluded if they presented with: (1) severe cognitive impairment or psychiatric disorders; (2) severe systemic conditions precluding oral examination; (3) acute oral infections or oral malignancies; or (4) incomplete questionnaire or oral examination data.

The sample size was calculated using G*Power software (version 3.1.9.7). Based on previous study evaluating oral conditions in relation to xerostomia [11, 12], the prevalence of oral diseases showed a significant difference between the xerostomia and non-xerostomia groups, corresponding to a Cohen’s h effect size of 0.414 (representing an approximate 20% absolute difference in prevalence between groups). To detect this difference using a two-tailed two-proportion Z-test with a significance level (*α*) of 5% and a statistical power (1 - *β*) of 95%, a minimum initial sample size of 303 participants was required. To account for the cluster sampling design, a design effect (*Deff*) of 1.5 was applied, which increased the required sample size to 455. Factoring in an anticipated 15% non-response or incomplete questionnaire rate, the final targeted sample size was determined to be approximately 535. Ultimately, a total of 583 participants completed the study with fully valid data, ensuring adequate power for cluster-adjusted analyses.

### Study variables

All participants underwent a comprehensive assessment consisting of a structured questionnaire and a subsequent clinical oral examination. The questionnaire collected information on socio-demographic characteristics, including age, gender, education level, and monthly income (Chinese Yuan, RMB); lifestyle factors, including smoking status and alcohol consumption; and health-related variables, including body mass index (BMI), sleep quality, self-rated health, and diabetes status. The standardized questionnaire was administered on-site by trained investigators, and standardized instructions were provided to assist participants in completing the survey. Sleep quality was assessed using a single-item self-reported sleep difficulty question referring to the previous month: “During the past month, how often did you have difficulty falling asleep, staying asleep, or waking up too early?” Responses were categorized into three groups according to symptom frequency: never, occasionally, and frequently.

Xerostomia was assessed using the Xerostomia Inventory (XI) [13], an 11-item instrument scored on a five-point Likert scale ranging from 1 (“Never”) to 5 (“Very often”), yielding a total score ranging from 11 to 55. Higher scores indicated greater xerostomia symptom severity. For descriptive analyses, participants were categorized into four severity levels according to XI scores: 11–22 points (no xerostomia), 23–33 points (mild xerostomia), 34–44 points (moderate xerostomia), and 45–55 points (severe xerostomia). For multivariable logistic regression analyses, participants with XI scores of 11–22 were classified as the non-xerostomia group, whereas those with scores ≥ 23 were classified as the xerostomia group. Oral health-related quality of life (OHRQoL) was assessed using the Geriatric Oral Health Assessment Index (GOHAI) [14], a 12-item instrument evaluating three domains: physical function, psychosocial function, and pain or discomfort. Higher GOHAI scores indicated better oral health-related quality of life.

All oral examinations were performed by two trained and calibrated dentists (κ > 0.80) using plane mouth mirrors and Community Periodontal Index (CPI) probes according to World Health Organization criteria [15]. Clinical oral assessments included dental caries experience, missing teeth, prosthetic restoration status, and periodontal status. Dental caries experience was recorded separately as the number of decayed teeth (DT), missing teeth (MT), and filled teeth (FT). To avoid potential overlap between the composite DMFT index and tooth loss, DT, MT, and FT were analyzed as separate variables in subsequent statistical analyses.

Prosthetic restoration status of missing teeth was stratified into three mutually exclusive categories: intact dentition (no missing teeth), unrestored missing teeth (presence of tooth loss without replacement), and restored missing teeth (tooth loss managed with fixed or removable prosthetic restorations). Periodontal status was assessed using the CPI framework, with scores assigned based on the severity of gingival bleeding, supragingival calculus, and periodontal pocket depth. Participants with a maximum CPI score ≥ 3 (indicating a probing pocket depth of ≥ 4 mm) were classified as having periodontitis. Given that the CPI is primarily a screening tool rather than a definitive full-mouth diagnostic measure for periodontitis, this threshold-based approach was adopted to balance clinical relevance with feasibility in large-scale community-based epidemiological examinations.

### Statistical analysis

Categorical variables were presented as frequencies and percentages, whereas continuous variables were expressed as medians and interquartile ranges (IQRs) due to their non-normal distribution. Differences in clinical and socio-demographic characteristics between the xerostomia and non-xerostomia groups were evaluated using Pearson’s chi-squared test for categorical variables and the Mann-Whitney U test for non-parametric continuous variables.

For logistic regression analyses, participants with Xerostomia Inventory (XI) scores of 11–22 were classified as the non-xerostomia group, whereas those with XI scores ≥ 23 were classified as the xerostomia group. Univariable and multivariable logistic regression analyses were performed to evaluate the independent associations between oral health indicators and xerostomia status. The adjusted model included age, education level, monthly income, sleep quality, self-rated health, diabetes, decayed teeth (DT), missing teeth (MT), filled teeth (FT), periodontitis, and prosthetic restoration of missing teeth as covariates.

Univariable and multivariable linear regression analyses were conducted to examine the associations between oral health indicators and oral health-related quality of life (GOHAI scores). In the multivariable model, adjustments were made for age, education level, monthly income, sleep quality, self-rated health, diabetes, decayed teeth (DT), missing teeth (MT), filled teeth (FT), periodontitis, prosthetic restoration of missing teeth, and xerostomia status.

To avoid conceptual overlap and potential multicollinearity between tooth loss and the composite DMFT index, DT, MT, and FT were analyzed as separate variables rather than using the aggregate DMFT score.

Receiver operating characteristic (ROC) curve analysis was performed as an exploratory assessment of the discriminative performance of the combined oral health indicators for distinguishing participants with and without xerostomia symptoms. Predicted probabilities derived from the fully adjusted logistic regression model were used to construct the ROC curve, and sensitivity, specificity, and the area under the curve (AUC) were calculated. This ROC analysis was not intended to evaluate the clinical diagnostic performance of the Xerostomia Inventory itself, but rather to evaluate the exploratory classification capacity of the combined oral health-related indicators in relation to symptom status.

All statistical analyses were performed using IBM SPSS Statistics version 27.0 (IBM Corp., Armonk, NY, USA) and R version 4.1.3 (R Foundation for Statistical Computing, Vienna, Austria). A two-sided P value < 0.05 was considered statistically significant.

## Results

A total of 583 older adults were included in the study, with an average age of 71 years old (SD = 6.4), comprising 330 females (56.6%) and 253 males (43.4%). Of these participants, 296 (50.8%) were classified as xerostomia, including 229 (39.3%) with mild xerostomia, 66 (11.3%) with moderate xerostomia, and 1 (0.2%) with severe xerostomia. Detailed XI scores and responses to individual items are presented in Table 1.

**Table 1:** Full data of XI and responses to individual items

Items	Never	Hardly never	Occasionally	Fairly often	Very often
My mouth feels dry.	152 (26.1)	114 (19.6)	164 (28.1)	115 (19.7)	38 (6.5)
My mouth feels dry when I am chewing food.	210 (36.0)	138 (23.7)	141 (24.2)	84 (14.4)	10 (1.7)
I wake up at night to drink water or other liquids.	195 (33.4)	135 (23.2)	131 (22.5)	99 (17.0)	23 (3.9)
I have difficulty eating dry food.	225 (38.6)	155 (26.6)	136 (23.3)	61 (10.5)	6 (1.0)
I have difficulty swallowing certain foods.	283 (48.5)	157 (26.9)	104 (17.8)	36 (6.2)	3 (0.5)
I need to drink liquids when I am swallowing food.	271 (46.5)	120 (20.6)	121 (20.8)	65 (11.1)	6 (1.0)
I need to suck sweets or similar to relieve the dry mouth sensation.	335 (57.5)	126 (21.6)	83 (14.2)	37 (6.3)	2 (0.3)
The skin on my face is dry.	234 (40.1)	180 (30.9)	137 (23.5)	30 (5.1)	2 (0.3)
My eyes are dry.	159 (27.3)	112 (19.2)	146 (25.0)	118 (20.2)	48 (8.2)
My lips are dry.	223 (38.3)	180 (30.9)	121 (20.8)	54 (9.3)	5 (0.9)
The inside of my nose feels dry.	277 (47.5)	166 (28.5)	106 (18.2)	31 (5.3)	3 (0.5)

*Abbreviations*: *XI* Xerostomia inventory

Data are presented as frequencies and percentages, n (%). The Xerostomia Inventory (XI) is an 11-item self-reported instrument. Each item is scored on a 5-point Likert scale: 1 = Never, 2 = Hardly ever, 3 = Occasionally, 4 = Fairly often, and 5 = Very often, yielding a total score ranging from 11 to 55, where higher scores indicate greater symptom severity

**Fig. 1:**
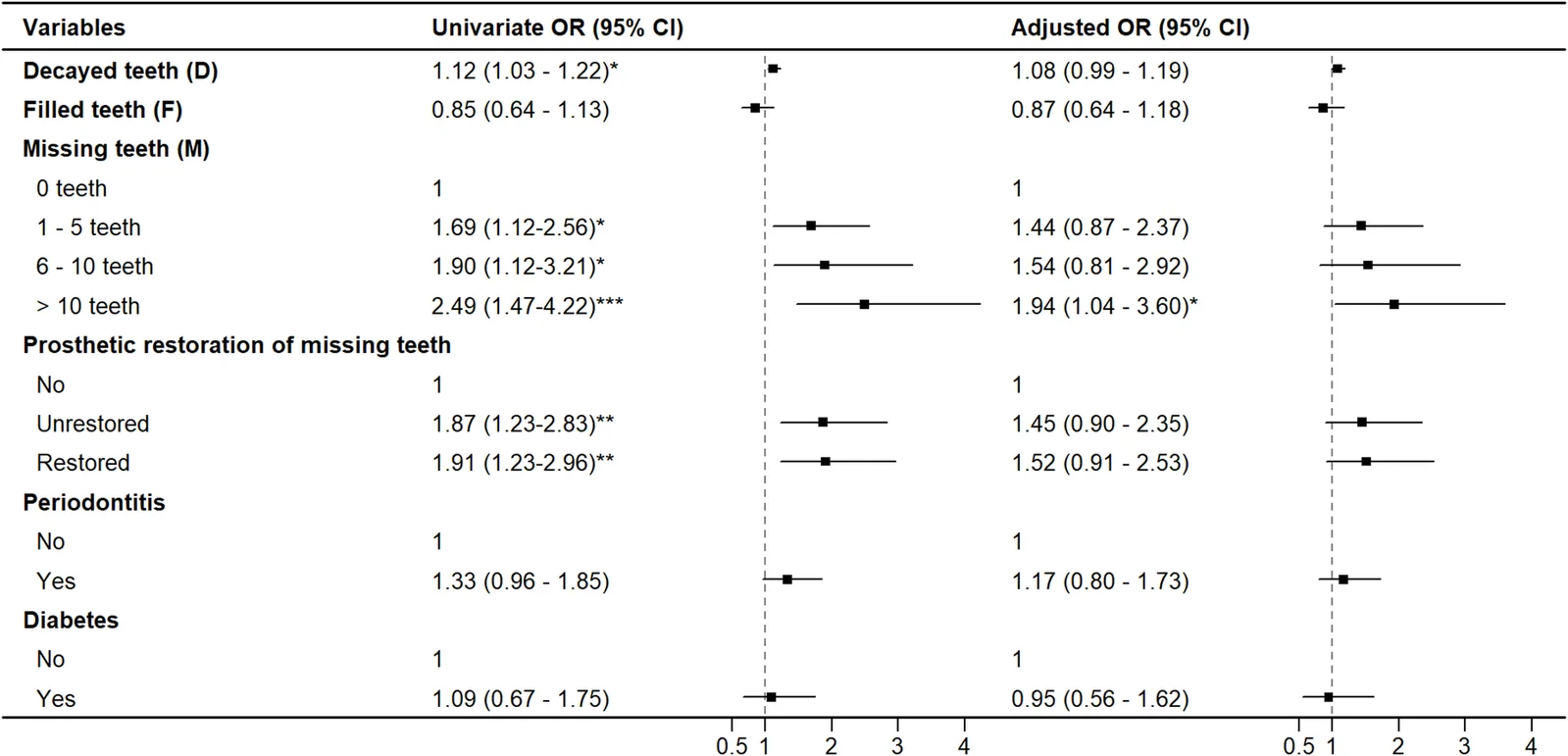
Associations of oral conditions with xerostomia according to unadjusted and adjusted logistic regression models. Abbreviations: GOHAI, General Oral Health Assessment Index. Adjusted model: further adjusted for age, education level, monthly income, sleep quality, self-rated health, decayed teeth, filled teeth, missing teeth, periodontitis, prosthetic restoration of missing teeth and diabetes. *p < .05; **p < .01; ***p < .001

For descriptive purposes, continuous XI scores are categorized into four severity levels based on equal score intervals: 11–22 points (no xerostomia), 23–33 points (mild xerostomia), 34–44 points (moderate xerostomia), and 45–55 points (severe xerostomia). For subsequent comparative and logistic regression analyses (as presented in Table 2 and Fig. 1), participants are dichotomized into the non-xerostomia group (11–22 points) and the xerostomia group (≥23 points) to distinguish individuals with any degree of dry mouth symptoms from those without

The demographic, health-related, and oral health characteristics of the study population according to xerostomia status are shown in Table 2. Compared with participants without xerostomia, those with xerostomia were more likely to be older (*p* = 0.006), have lower educational attainment (*p* = 0.004), lower monthly income (*p* = 0.003), poorer sleep quality (*p* < 0.001), and worse self-rated health (*p* < 0.001). No significant difference was observed in the prevalence of diabetes between participants with and without xerostomia (13.9% vs. 12.9%, *p* = 0.743).

**Table 2:** Characteristics of the study population according to xerostomia status

Variables	Total (*N* = 583)	No xerostomia (*n* = 287)	Xerostomia (*n* = 296)	P*
Gender				0.081
Male	253 (43.4)	135 (47.0)	118 (39.9)	
Female	330 (56.6)	152 (53.0)	178 (60.1)	
Age				
55 to 70 years	291 (49.9)	160 (55.7)	131 (44.3)	0.006
Over 70 years	292 (50.1)	127 (44.3)	165 (55.7)	
Education level				0.004
Primary school	437 (75.0)	200 (69.7)	237 (80.1)	
> Primary school	146 (25.0)	87 (30.3)	59 (19.9)	
Monthly income				0.003
≤ 1000	447 (76.7)	205 (71.4)	242 (81.8)	
> 1000	136 (23.3)	82 (28.6)	54 (18.2)	
BMI				0.95
Normal	225 (38.6)	127 (44.3)	128 (43.2)	
Overweight	234 (40.1)	115 (40.1)	119 (40.2)	
Obesity	94 (16.1)	45 (15.7)	49 (16.6)	
Smoking status				0.316
No	459 (78.7)	221 (77.0)	238 (80.4)	
Yes	124 (21.3)	66 (23.0)	58 (19.6)	
Alcohol consumption				0.207
No	434 (74.4)	207 (72.1)	227 (76.7)	
Yes	149 (25.6)	80 (27.9)	69 (23.3)	
Sleep Quality				< 0.001
Never	251 (43.1)	152 (53.0)	99 (33.4)	
Occasionally	205 (35.2)	86 (30.0)	119 (40.2)	
Frequently	127 (21.8)	49 (17.1)	78 (26.4)	
Self-rated health				< 0.001
Poor	104 (17.8)	44 (15.3)	60 (20.3)	
Acceptable	247 (42.4)	92 (32.1)	155 (52.4)	
Good	232 (39.8)	151 (52.6)	81(27.4)	
Diabetes				
No	505 (86.6)	250 (87.1)	255 (86.1)	0.743
Yes	78 (13.4)	37 (12.9)	41 (13.9)	
Periodontitis				0.090
No	337 (57.8)	176 (61.3)	161 (54.4)	
Yes	246 (42.2)	111 (38.7)	135 (45.6)	
Decayed teeth (D)	1.0 (0.0, 2.0)	0.0 (0.0, 2.0)	1.0 (0.0, 3.0)	0.007
Filled teeth (F)	0.0 (0.0, 0.0)	0.0 (0.0, 0.0)	0.0 (0.0, 0.0)	0.199
Missing teeth (M)	2.0 (0.0, 6.0)	2.0 (0.0, 5.0)	3.0 (1.0, 8.0)	0.009
0 teeth	146 (25.0)	89 (31.0)	57 (19.3)	0.004
1–5 teeth	248 (42.5)	119 (41.5)	129 (43.6)	
6–10 teeth	93 (16.0)	42 (14.6)	51 (17.2)	
> 10 teeth	96 (16.5)	37 (12.9)	59 (19.9)	
Prosthetic restoration of missing teeth				0.004
No	146 (25.0)	89 (31.0)	57 (19.3)	
Unrestored	248 (42.5)	113 (39.4)	135 (45.6)	
Restored	189 (32.4)	85 (29.6)	104 (35.1)	
GOHAI	52.0 (46.0, 57.0)	56.0 (51.0, 58.0)	51.0 (45.0, 55.0)	< 0.001

Data are numbers (percentages) unless otherwise indicated

*Abbreviations*: *BMI* Body mass index, *GOHAI* General oral health assessment index

*Simple differences between participants with or without xerostomia were assessed by a nonparametric test (for continuous variables) or chi-square test (for categorical variables)

Differences in oral health indicators were also observed according to xerostomia status. Participants with xerostomia had a higher median number of decayed teeth [1.0 (IQR: 0.0, 3.0) vs. 0.0 (IQR: 0.0, 2.0), *p* = 0.007] and missing teeth [3.0 (IQR: 1.0, 8.0) vs. 2.0 (IQR: 0.0, 5.0), *p* = 0.009] than those without xerostomia, whereas no significant difference was observed in the number of filled teeth (*p* = 0.199). Furthermore, a greater proportion of participants with xerostomia had more than 10 missing teeth compared with those without xerostomia (19.9% vs. 12.9%, *p* = 0.004). Regarding prosthetic restoration status, participants with xerostomia were significantly less likely to have all missing teeth restored and more likely to remain unrestored (*p* = 0.004). Additionally, the xerostomia group demonstrated significantly lower GOHAI scores compared to the non-xerostomia group [51.0 (IQR: 45.0–55.0) vs. 56.0 (IQR: 51.0–58.0), *p* < 0.001], indicating a poorer oral health-related quality of life.

### Logistic regression analysis

Figure 1 presents the results of the binary logistic regression analyses examining the associations between clinical oral status parameters and xerostomia status, with xerostomia status (non-xerostomia [XI score 11–22] vs. xerostomia [XI score ≥ 23]) defined as the binary dependent variable. In the unadjusted model significant associations were observed for decayed teeth (DT), tooth loss categories, and prosthetic restoration status. Specifically, each additional decayed tooth was associated with a 12% increase in the odds of having xerostomia (OR = 1.12, 95% CI: 1.03–1.22, *p* = 0.011). For tooth loss, a clear gradient was noted; compared with participants without tooth loss, those with 1–5 missing teeth (OR = 1.69, 95% CI: 1.12–2.56, *p* = 0.013), 6–10 missing teeth (OR = 1.90, 95% CI: 1.12–3.21, *p* = 0.017), and more than 10 missing teeth (OR = 2.49, 95% CI: 1.47–4.22, *p* < 0.001) all exhibited significantly greater odds of experiencing xerostomia. Regarding prosthetic restoration status of missing teeth, using individuals with no missing teeth as the reference group, both participants with unrestored missing teeth (OR = 1.87, 95% CI: 1.23–2.83, *p* = 0.003) and those with restored missing teeth (OR = 1.91, 95% CI: 1.23–2.96, *p* = 0.004) demonstrated significantly higher odds of having xerostomia.

After adjusting for potential confounding variables—including age, educational level, monthly income, sleep quality, self-rated health, decayed teeth, filled teeth, missing teeth, periodontitis, prosthetic restoration, and diabetes—the associations for decayed teeth and prosthetic status were attenuated and lost statistical significance (all *p* > 0.05). Remarkably, the association between severe tooth loss and xerostomia remained statistically robust. Compared with participants without tooth loss, those missing more than 10 teeth still exhibited a near twofold increase in the odds of having xerostomia (adjusted OR = 1.94, 95% CI: 1.04–3.60, *p* = 0.037). Furthermore, a dose - response pattern was consistently observed across both models, whereby higher levels of tooth loss were progressively associated with greater odds of xerostomia, demonstrated by the rising adjusted odds ratios from 1.44 (95% CI: 0.87–2.37) for 1–5 missing teeth, to 1.54 (95% CI: 0.81–2.92) for 6–10 missing teeth, and peaking at 1.94 for the most severe category.

### Linear regression analysis

Table 3 presents the results of the linear regression analyses examining the associations between clinical oral health indicators and GOHAI scores, with the total GOHAI score treated as the continuous dependent variable representing oral health-related quality of life (OHRQoL).

**Table 3:** Associations of oral conditions with GOHAI scores according to unadjusted and adjusted linear regression models

Variables	Univariate			Adjusted		
	B ± SE	β	*p*	B ± SE	β	*p*
Decayed teeth(D)	-0.27 ± 0.39	-0.07	0.091	-0.08 ± 0.14	-0.02	0.565
Filled teeth(F)	0.65 ± 0.56	0.05	0.243	0.07 ± 0.47	0.05	0.883
Missing teeth(M)						
0 teeth	1			1	1	
1–5 teeth	-1.79 ± 0.79 *	-0.11	0.023	-0.71 ± 0.77	-0.05	0.355
6–10 teeth	-4.15 ± 1.00 ***	-0.20	< 0.001	-2.80 ± 0.99 **	-0.13	0.005
> 10 teeth	-5.44 ± 0.99 ***	-0.26	< 0.001	-3.33 ± 0.95 ***	-0.16	< 0.001
Periodontitis						
No	1			1	1	
Yes	-0.81 ± 0.65	-0.05	0.216	0.22 ± 0.59	0.01	0.716
Prosthetic restoration of missing teeth						
No	1			1	1	
Unrestored	-2.18 ± 0.80 **	-0.14	0.007	-0.12 ± 0.74	-0.01	0.876
Restored	-2.73 ± 0.85 **	-0.17	0.001	-0.52 ± 0.79	-0.03	0.515
Xerostomia						
No	1			1	1	
Yes	-7.97 ± 0.55 ***	-0.51	< 0.001	-7.05 ± 0.58 ***	-0.45	< 0.001
Diabetes						
No	1			1		
Yes	0.84 ± 0.95	0.04	0.375	0.71 ± 0.83	0.03	0.389

Adjusted model: further adjusted for age, education level, monthly income, sleep quality, self-rated health, decayed teeth, filled teeth, missing teeth, periodontitis, prosthetic restoration of missing teeth, xerostomia and diabetes

*Abbreviations*: *SE* Standard error, *GOHAI* General oral health assessment Index

*p < .05; **p < .01; ***p < .001

In the unadjusted model, significant negative associations with GOHAI scores were observed for tooth loss, prosthetic status, and xerostomia. Compared with participants retaining all natural teeth, those with missing teeth had lower GOHAI scores, indicating poorer OHRQoL. A dose-response pattern was observed, with progressively lower GOHAI scores associated with increasing levels of tooth loss: 1–5 missing teeth (B = -1.79, SE = 0.79, 95% CI: -3.34 to -0.24, *p* = 0.023), 6–10 missing teeth (B = -4.15, SE = 1.00, 95% CI: -6.11 to -2.19, *p* < 0.001), and more than 10 missing teeth (B = -5.44, SE = 0.99, 95% CI: -7.38 to -3.50, *p* < 0.001). Regarding prosthetic restoration status of missing teeth, using participants without tooth loss as the reference group, both unrestored missing teeth (B = -2.18, SE = 0.80, 95% CI: -3.75 to -0.61, *p* = 0.007) and restored missing teeth (B = -2.73, SE = 0.85, 95% CI: -4.40 to -1.06, *p* = 0.001) were associated with lower GOHAI scores. Xerostomia exhibited the strongest unadjusted association with GOHAI scores, corresponding to a reduction of 7.97 points (B = -7.97, SE = 0.55, 95% CI: -9.05 to -6.89, *p* < 0.001).

After adjustment for age, educational level, monthly income, sleep quality, self-rated health, decayed teeth, filled teeth, missing teeth, periodontitis, prosthetic restoration, xerostomia, and diabetes, the associations for prosthetic restoration status and mild tooth loss (1–5 teeth) were attenuated and no longer statistically significant (all *p* > 0.05). However, severe tooth loss and xerostomia remained independently associated with lower GOHAI scores. Compared with participants without tooth loss, those with 6–10 missing teeth (B = -2.80, SE = 0.99, 95% CI: -4.74 to -0.86, *p* = 0.005) and more than 10 missing teeth (B = -3.33, SE = 0.95, 95% CI: -5.19 to -1.47, *p* < 0.001) continued to have significantly lower GOHAI scores, suggesting a dose - response relationship between the extent of tooth loss and OHRQoL. In addition, xerostomia remained strongly associated with lower GOHAI scores in the fully adjusted model (B = -7.05, SE = 0.58, 95% CI: -8.19 to -5.91, *p* < 0.001).

### Area under curve

**Fig. 2:**
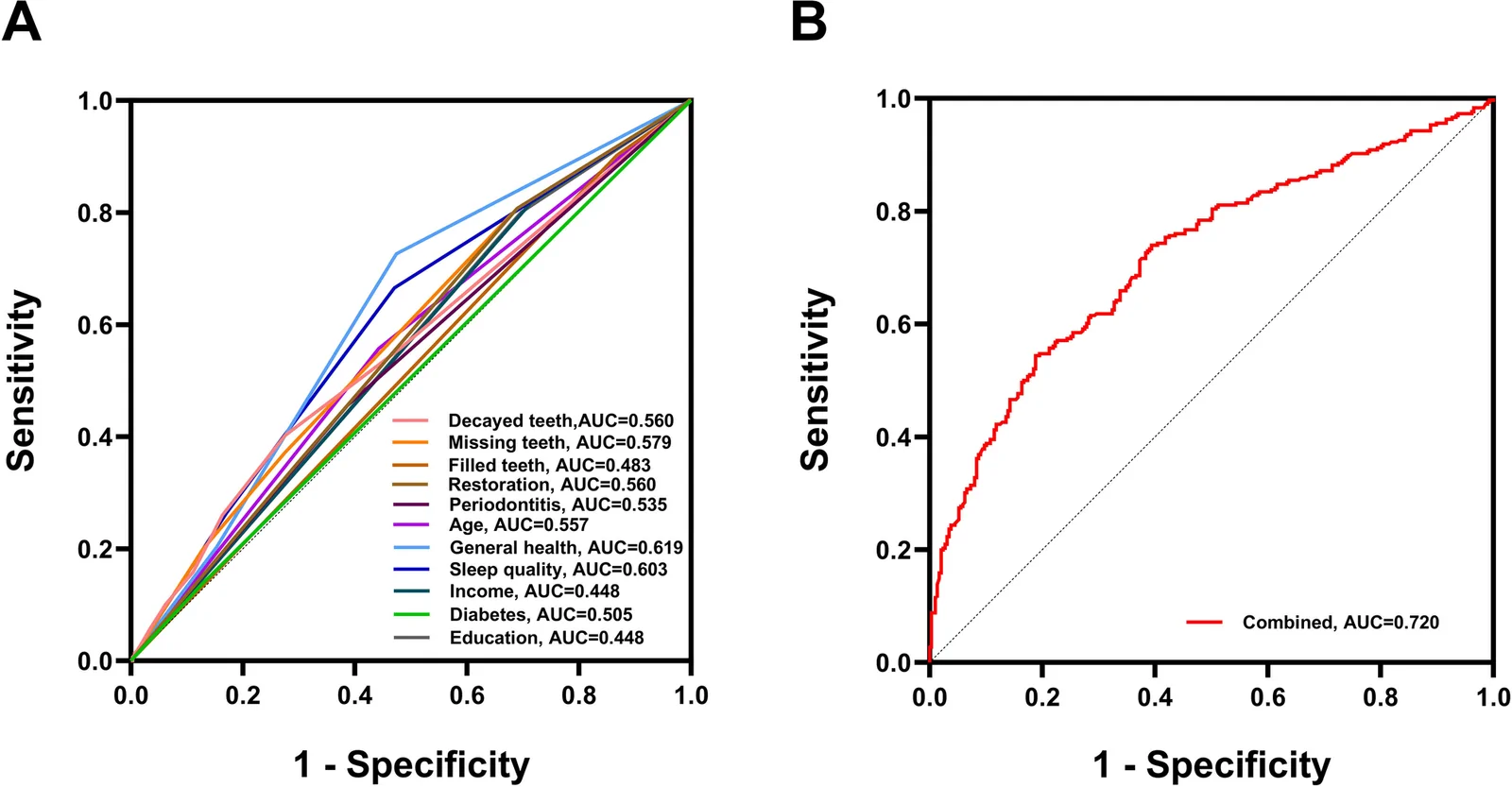
Predicted probability of the independent parameters and combined predictors for xerostomia. **A** Predicted probability of the independent parameters. **B** Predicted probability of the combined predictors. AUC: area under the curve

Figure 2 presents the exploratory receiver operating characteristic (ROC) curve analysis evaluating the classification performance of the combined indicators in distinguishing participants with and without xerostomia symptoms. The combined analysis incorporated socio-demographic characteristics (age, education level, income), health-related covariates (sleep quality, self-rated health, and diabetes), and the individual clinical oral components (decayed teeth, filled teeth, missing teeth, periodontitis and prosthetic restoration status).

The results indicated that while each single parameter exhibited limited discriminatory capacity when analyzed in isolation, the fully combined exploratory model demonstrated an acceptable-to-moderate discriminative performance, yielding an updated area under the curve (AUC) of 0.720. This indicates that the combination of these multi-dimensional indicators provides modest exploratory information for classifying symptom-defined xerostomia groups within this study population, rather than serving as a clinically definitive diagnostic or predictive tool.

## Discussion

In this study, we investigated the associations among distinct clinical oral health indicators, xerostomia, and oral health-related quality of life (OHRQoL) in a community-dwelling older population after adjustment for relevant sociodemographic and health-related factors. Severe tooth loss was independently associated with higher odds of xerostomia, with a statistically robust association observed specifically among participants missing more than 10 teeth. Furthermore, xerostomia and advanced tooth loss (≥ 6 missing teeth) remained independently associated with lower GOHAI scores, indicating poorer OHRQoL. In contrast, other clinical oral health indicators, including decayed teeth, filled teeth, periodontitis, and prosthetic restoration status, were not independently associated with xerostomia or OHRQoL after full adjustment.

In line with the main findings, we further explored potential explanations for the observed association between tooth loss and xerostomia. The odds of experiencing xerostomia increased progressively with the severity of natural tooth loss, consistent with epidemiological patterns reported by Stankeviciene et al. [16]. Previous studies have similarly shown that individuals with self-perceived salivary hypofunction possess significantly fewer remaining natural teeth and poorer overall dental status [17]. The potential for reverse causality must be carefully acknowledged. Biologically, chronically reduced salivary flow and altered salivary composition heavily impair the oral cavity’s physiological self-cleansing mechanisms, buffering capacity, and antimicrobial defense [18–21]. This disruption facilitates biofilm stagnation and microbiome dysbiosis, which can accelerate the cumulative oral disease burden—such as rampant dental caries and advanced periodontal attachment loss—ultimately culminating in permanent tooth extractions over time [22, 23]. Recent high-quality evidence further corroborates these mechanistic links, demonstrating that salivary dysfunction, microbiome alterations, and cumulative oral disease burden are interrelated and may be exacerbated in both community-dwelling and radiotherapy-exposed older populations [24].

Decayed teeth demonstrated positive associations with xerostomia in univariable models, which aligns with observations in previous literature. However, these relationships were attenuated and lost statistical significance after full multivariable adjustment. This lack of independent significance warrants deeper critical interpretation and suggests that tooth loss may serve as a cumulative indicator of lifetime oral health deterioration rather than reflecting current active disease. In an aging population with a mean age of 71.0 years, active dental caries and filled surfaces frequently represent transient or historical stages of chronic oral pathology. Over a prolonged lifespan, severely affected teeth may have already been lost prior to assessment, a phenomenon heavily aligned with historical evidence that individuals with severe xerostomia often experience accelerated tooth loss progressing to advanced edentulism at an earlier age [22, 25]. In addition, our study found that participants with xerostomia tended to have lower educational and income. These factors may limit access to preventive dental care and increase reliance on therapeutic extractions over restorative interventions. This aligns with recent evidence linking neighborhood deprivation and lower-quality living environments to higher prevalence of tooth loss and chronic oral dryness among older adults [26].

Interestingly, prosthetic restoration of missing teeth was not independently associated with xerostomia after full multivariable adjustment. Previous studies have reported inconsistent findings regarding the relationship between prosthetic rehabilitation and xerostomia. Some investigations have shown that individuals wearing removable dentures are more likely to report xerostomia symptoms and reduced salivary flow [17, 27]. Adequate saliva plays an important role in maintaining adhesion, cohesion, and surface tension between dentures and oral tissues, thereby contributing to denture retention and comfort [28]. Consequently, xerostomia may adversely affect denture function and patient satisfaction. Conversely, other studies have suggested that fixed or implant-supported prosthetic rehabilitation may be associated with fewer xerostomia-related complaints and improved oral comfort [29]. which observed an improvement of xerostomia after restoration of missing teeth. The lack of association observed in the present study may partly reflect the relatively broad categorization of prosthetic restoration status. Information regarding prosthesis type, quality, duration of use, and patient adaptation was not available. Therefore, potentially important differences between removable, fixed, and implant-supported restorations could not be evaluated. Future longitudinal studies incorporating detailed prosthetic characteristics are warranted to further clarify how different prosthetic restorations are associated with xerostomia among older adults.

Several underlying biological and physiological processes have been implicated in mediating the associations between xerostomia and oral health outcomes. Previous studies have reported that impaired salivary function is associated with a higher prevalence of dental erosion [8, 10], as saliva plays a critical role in self-cleansing, antimicrobial activity, buffering of oral pH, and enamel remineralization. Importantly, xerostomia may reflect not only reduced salivary quantity but also alterations in salivary composition, which may influence the subjective perception of oral dryness independently of salivary flow rate [30]. Alterations in the oral microbiota have also been suggested as a potential mechanism linking xerostomia with adverse oral conditions [16, 31, 32]. Reduced salivary function may be associated with less effective microbial clearance and lower concentrations of salivary immune components, thereby facilitating microbial aggregation and prolonged biofilm persistence. Such changes have been linked to a higher prevalence of dental caries, periodontitis, and halitosis in observational studies [33]. Recent studies have further highlighted the role of salivary dysfunction and oral microbial imbalance in shaping oral health outcomes, supporting the biological plausibility of the associations observed in the present study [34]. In addition, age-related gingival recession and root surface exposure may further increase susceptibility to dental erosion among older adults [35]. In the present study, after decomposing the DMFT index into its individual components (decayed teeth, filled teeth, and missing teeth), neither decayed teeth nor filled teeth remained independently associated with xerostomia after multivariable adjustment. This finding may reflect the different clinical meanings represented by the three components of the DMFT index. Decayed and filled teeth generally represent current or previous disease activity, whereas missing teeth reflect the cumulative consequence of long-term oral disease. In an older population, substantial proportions of teeth affected by severe caries or other oral conditions may already have been extracted. Consequently, when missing teeth categories are simultaneously included in fully adjusted models, part of the variation previously captured by decayed or filled teeth may be accounted for by tooth loss, attenuating their independent associations with xerostomia.

The relationship between xerostomia and periodontal disease remains inconclusive in the literature. This uncertainty is reflected in our study, where no independent significant association was observed in multivariable analyses. Hirotomi et al. [36] reported that lower salivary flow was associated with impaired periodontal tissues, potentially facilitating opportunistic microbial colonization. Animal studies have observed links between experimental periodontitis and altered salivary gland function, with partial recovery following resolution of periodontal inflammation [37]. Similarly, clinical observations indicate that adults with chronic periodontitis may exhibit reduced salivary flow, possibly reflecting local inflammatory influences on glandular secretion [38]. Prospective evaluations among patients with suspected xerostomia have also noted higher plaque indices and increased bleeding on probing compared to controls [39]. However, other investigation assessing multiple periodontal parameters—including plaque, bleeding, calculus indices, probing depth, and attachment levels—found no statistically significant differences between xerostomia patients and non-affected individuals [40]. Taken together, no significant independent association was detected between periodontal status, as assessed by the Community Periodontal Index (CPI), and xerostomia. While the CPI provides an efficient tool for large-scale epidemiological screening, it examines specific index teeth rather than full-mouth, six-site clinical charting, potentially leading to underestimation or misclassification of periodontal disease severity in our cohort. Given the cross-sectional design, these associations should be interpreted as correlational rather than causal, and caution is warranted when drawing inferences regarding xerostomia and periodontal health.

In the present study, severe tooth loss and xerostomia emerged as the principal oral health conditions independently associated with poorer oral health-related quality of life (OHRQoL) among community-dwelling older adults. Previous studies have suggested that maintaining a higher number of retained natural teeth is conceptually linked to superior masticatory efficiency, which supports adequate nutritional intake and overall systemic well-being [41]. Participants with greater numbers of missing teeth tended to exhibit lower GOHAI scores, a finding consistent with previous evidence showing that OHRQoL declines significantly among older individuals with more than five missing teeth [42]. Given the cross-sectional design of this study, these observed associations likely reflect the cumulative functional burden of long-term oral status and salivary impairment, rather than direct, unidirectional causation. However, no significant independent association was observed between prosthetic restoration status and OHRQoL after multivariable adjustment. This finding should be interpreted cautiously, as information regarding prosthesis type and quality was not available, and different forms of prosthetic rehabilitation may have distinct relationships with OHRQoL. A previous study revealed a significant negative impact of a lower number of teeth/implants in the upper jaw and the presence of gum-supported dentures in both jaws on GOHAI scores [43]. Consequently, combining these prosthetic restoration modalities into a single ‘restored’ category may have attenuated their distinct effects on OHRQoL in our final models. Notably, xerostomia demonstrated one of the strongest independent associations with lower GOHAI scores in the fully adjusted model. This robust inverse relationship is well-aligned with established literature, as clinical manifestations such as difficulties in chewing, swallowing, speaking, and maintaining oral comfort are known to heavily intertwine the physiological presentation of xerostomia with the functional and psychological dimensions of quality of life [44]. Similar detrimental associations between xerostomia and quality-of-life outcomes have also been reported in clinical populations with salivary gland dysfunction, including patients receiving head and neck radiotherapy [45]. Taken together, these findings underscore the importance of severe tooth loss and xerostomia as key oral health conditions associated with reduced OHRQoL in community-dwelling older adults.

However, some limitations need to be taken into account. First, the cross-sectional design of this study inherently precludes drawing causal inferences or establishing temporal relationships among tooth loss, xerostomia, and oral health-related quality of life (OHRQoL). Second, xerostomia status was determined solely using the Xerostomia Inventory (XI), a validated self-reported instrument that reflects subjective dry mouth symptoms but does not directly quantify physiological salivary output. Objective measures of salivary flow (e.g., unstimulated and stimulated salivary flow rates) were unavailable, and symptom definitions may vary across studies, potentially contributing to measurement heterogeneity. Third, although multiple sociodemographic, health-related, and oral health variables were adjusted for, residual confounding from unmeasured factors remains possible. Most notably, detailed histories of medication use and polypharmacy—which represent paramount determinants of subjective dry mouth symptoms due to the anticholinergic or xerogenic side effects of commonly prescribed geriatric drugs (e.g., certain antihypertensives, diuretics, and antidepressants) were not comprehensively captured due to logistical field constraints during the initial community screenings. Similarly, other systemic or autoimmune comorbidities that directly compromise salivary gland parenchyma, such as Sjögren’s syndrome, and lifestyle factors (e.g., diet, oral hygiene practices) were not captured, warranting cautious interpretation of the independent oral-status associations. Fourth, the periodontal assessment using the Community Periodontal Index (CPI), while standardized, may underestimate the true extent of periodontal burden compared with full-mouth clinical probing protocols. In addition, the lack of assessment for other oral conditions (e.g., occlusion, removable prosthesis) might limit the comprehensiveness of our findings. Fifth, health-related covariates (such as sleep quality, self-rated general health, and lifestyle habits) alongside the XI and GOHAI outcome dimensions were captured via structured face-to-face subjective questionnaires. While appropriate for assessing subjective symptoms, these self-reported metrics might introduce recall and social desirability biases. Finally, the ROC analysis was performed as an exploratory assessment of classification performance rather than to develop or validate a clinical diagnostic model. The observed AUC of 0.720 indicates acceptable-to-moderate discrimination and should not be interpreted as evidence of clinical diagnostic utility. Considering these methodological boundaries, future multicenter, prospective cohort studies with larger heterogeneous samples, objective salivary flow measurements, comprehensive medication profiling, and advanced microbiome assessments are warranted to further elucidate the complex associations among oral disease burden, xerostomia, and quality of life in older adults.

## Conclusion

In conclusion, xerostomia was prevalent in this community-dwelling elderly population and was independently associated with greater tooth loss. Both xerostomia and advanced tooth loss were linked to lower oral health-related quality of life. These findings highlight the importance of considering tooth loss and xerostomia as key oral health conditions associated with compromised OHRQoL among older adults. Comprehensive oral health assessment and appropriate management of oral conditions may therefore be beneficial in promoting well-being in aging populations.

## Supplementary Information

Supplementary Material 1.

## Data Availability

The datasets used and analyzed during the current study are available from the corresponding author on reasonable request.

## Abbreivations

OHRQoL: Oral health and quality of life
XI: Xerostomia inventory
GOHAI: Geriatric oral health assessment index
BMI: Body mass index
CPI: Community periodontal index
DMFT: Decayed, missing, and filled teeth index
DT: Decayed teeth
MT: Missing teeth
FT: Filled teeth
ROC: Receiver operating characteristic
AUC: Aea under the curve
SD: Standard deviation
CI: Confidence interval
SE: Standard error
